# Efficacy of Rotational Grazing on the Control of *Rhipicephalus microplus* Infesting Calves in Humid Tropical Conditions

**DOI:** 10.1155/2024/7558428

**Published:** 2024-10-15

**Authors:** Gabriel Cruz-González, Juan Manuel Pinos-Rodríguez, Miguel Ángel Alonso-Díaz, Dora Romero-Salas, Jorge Genaro Vicente-Martínez, Agustín Fernández-Salas, Jesús Jarillo-Rodríguez, Epigmenio Castillo-Gallegos

**Affiliations:** ^1^Faculty of Veterinary Medicine and Zootechnics, University of Veracruz, Veracruz 91710, Mexico; ^2^Center for Teaching, Research, and Extension in Tropical Livestock, Faculty of Veterinary Medicine and Zootechnics, National Autonomous University of Mexico, Mexico City 93600, Mexico

**Keywords:** cattle, continuous grazing, integrated pest management, rotational grazing, ticks

## Abstract

Grazing management through pasture rotation has been mentioned as a viable alternative for the control of *Rhipicephalus microplus*; however, there is limited information on rotational grazing at field level. The objectives of this research were (1) to evaluate the effect of rotational grazing with 30 and 45 days of pasture rest and continuous grazing (without rest) on *R. microplus* tick loads in Brahman heifers and its most common crosses with Holstein; (2) to know the parasite dynamics of *R. microplus* under the three grazing systems in the humid tropics of Mexico; and (3) to determine the effect of the treatments on the characteristics of the pastures (availability of forage biomass, plant height, and soil cover). The experiment was carried out for 1 year from April 2022 to March 2023, with three grazing treatments: Treatments 1 and 2 considered rotational grazing with 30 (RT30) and 45 days of rest (RT45), respectively, and Treatment 3 as continuous grazing (CT00). Thirty calves from 8 to 12 months of initial age were distributed in each treatment (*n* = 10). Every 14 days, the number of engorged and semiengorged female ticks in cattle was determined. Concomitantly, the region's temperature, relative humidity, and rainfall were recorded, as well as the agronomic characteristics of the pasture. Rotational grazing animals with 30 days of rest had the highest number of ticks, while grazing animals with 45 days of rest had the least. Tick load dynamics among groups did not correlate with climatic variables (*p* > 0.05). The height and grass cover presented significant changes due to grazing (*p* < 0.05), which could influence the tick cycle by the exposure of the soil, modifying the microclimatic conditions and consequently harming the survival and development of *R. microplus* in the evaluated systems. The negative effect of rotational grazing on the nonparasitic phase of *R. microplus* deserves further studies.

## 1. Introduction

Cattle farming is one of the most important economic activities worldwide, where tropical and subtropical regions play a significant role due to their large livestock inventory. These areas having have a high potential for grazing livestock; however, the prevailing climatic conditions are also favorable for the successful development of tick populations during almost the entire year [1]. Among these parasites, *Rhipicephalus microplus* stands out, which is considered the tick that most affects the livestock industry in the tropics and subtropics of the world. Furthermore, *R. microplus* is a potential transmitter of pathogens such as *Anaplasma marginale* and *Babesia* spp. [2]. The life cycle of this tick is short and monoxenic, which can favor the development of up to five generations per year [3], a situation that increases the need to apply control methods throughout the year [4, 5].

The application of chemical acaricides is the most used strategy, however, its frequent use has caused the contamination of ecosystems, residues in meat and milk, and has promoted the development of resistant ticks [6, 7]. Consequently, many efforts have been made to find alternative methods to control *R. microplus* such as the grazing management based on pasture rotation [5, 8, 9]. Epidemiologically, control through pasture rotation can greatly impact tick parasitism, since it is known that 95% of their populations are found in pastures and the rest in animals [10]. Appropriate grazing management will reduce the encounter phase between *R. microplus* larvae and cattle, which will cause the death of the larva or its weakening by starvation or desiccation. Intensive rotational grazing has shown to be a potential strategy to recover degraded pastures, reduce the intensity of methane production, and make production systems more efficient [11]. An essential aspect of these rotational systems is the determination of adequate pasture rest times for a significant control of *R. microplus* [5], since these may have different requirements through time according to the geoclimatic variability of the regions [9, 12, 13]. In this regard, in previous studies, short and long rest times in the paddocks have been used. Short times (20 days) [12] appear to have no effect on ticks on animals, and long times (more than 49 days) may decrease pasture quality [11]. Therefore, it is necessary to explore times of between 30 and 45 days considering the elimination of ticks, but also taking care of the nutritional quality of the pastures.

On the other hand, in livestock regions, various breeds of cattle and their crosses are managed, which makes it necessary to evaluate tick control under diverse conditions of crossbreeding of the animals and to have a better perspective on the potential use of paddock rotation under different zootechnical conditions.

The objectives of the present study were (1) to evaluate the effect of rotational grazing with 30 and 45 days of pasture rest and continuous grazing (without rest) on *R. microplus* tick loads in Brahman heifers and its most common crosses with Holstein; (2) to know the parasite dynamics of *R. microplus* under the three grazing systems in the humid tropics of Mexico; and (3) to describe the effect of the treatments on the characteristics of the pastures (availability of forage biomass, plant height, and soil cover).

## 2. Materials and Methods

### 2.1. Location

The experiment was carried out at the Centre for Teaching, Research, and Extension in Tropical Livestock (CEIEGT) of the Faculty of Veterinary Medicine and Zootechnics of the National Autonomous University of Mexico (20°02′ N, 97°06′ 0) [14], from April 2022 to March 2023.

### 2.2. Experiment Design

Three grazing management modalities were considered: (1) RT30, rotational grazing with 3 days of grazing and 30 days of rest, with 2 ha divided into 11 paddocks of 0.1818 ha each; (2) RT45, rotational grazing with 3 days of grazing and 45 days of rest, with 2 ha divided into 16 paddocks of 0.1250 ha each; and (3) CT00, continuous grazing with 2 ha without internal divisions and without rest periods. The three treatments had the same latitude, the same surface irregularities, and all with African star grass (*Cynodon nlemfuensis*). Historically, paddocks have only been used for cattle grazing in the last three decades until the start of the experiment. The pastures did not receive any antitick treatment nor were tick populations added to the paddocks before the experiment.

Thirty calves with an average initial weight and age of 143 ± 33 kg and 8 to 12 months, respectively, were used. Three groups of 10 animals were formed and randomly distributed to each treatment. Each group consisted of six Brahman (Zebu) calves, two 3/4 × 1/4 (Brahman × Holstein), one 7/8 × 1/8 (Brahman × Holstein), and one 5/8 × 3/8 (Holstein × Brahman). For the formation of the groups, apart from the genotype, they were also balanced according to coat color and weight. At the beginning of the study, the stocking rate managed per hectare for each treatment was 3.1 animal units (AU = 450 kg live weight).

### 2.3. Animal Management

During the entire experiment, daily, the calves were provided with 1 kg of concentrated feed per animal and water ad libitum. The three established treatments had no common areas; each group had mobile and exclusive drinkers and feeders. The animals were exposed to ticks ever since they were born until before the experiment, and they were sprayed regularly against ticks with pyrethroids and organophosphates. To have internal and external parasite-free animals at the beginning of the experiment, the heifers were treated 15 days before the start (Day 0) of the experiment against gastrointestinal parasites with albendazole and against ticks and flies with coumaphos. These chemical products were used because no resistance to them has been identified in the experimental station. During the study, the animals did not receive any acaricide treatment. However, they were under continuous medical evaluation to monitor tick loads and any health effects that might occur. The study was approved by the Bioethics and Animal Welfare Commission (number 015/21) of the Faculty of Veterinary Medicine and Zootechnics, University of Veracruz (UV-FMVZ).

### 2.4. Parasitic Dynamics of *R. microplus*

To determine the parasite loads in the calves, tick counts were carried out at 14-day intervals from 7:00 a.m. to 9:00 a.m., using a comprehension ramp, for a total of 26 counts in the year. *R. microplus* ticks with a length greater than 4.5 mm were registered without detaching them, according to the methodology validated by Miraballes, Taño, and Riet-Correa [15]. A peak was considered that point which count was larger or equal to 25 ticks, and the tendency of the line connecting it with the previous count was ascendent (positive slope), and that one connecting it with the following point was descendant (negative slope). Counts were always performed at the same time and by the same researcher.

### 2.5. Pasture Agronomic Variables

Forage biomass (leaf, stem, and dead matter), height, and forage cover were recorded every 28-day intervals on CT00, and before and after (RT30 and RT45) grazing of the calves. Three paddocks from RT30 and three from RT45 were randomly selected to obtain height and biomass (before and after cattle grazing). On each paddock, three representative sampling points (beginning, middle ,and end of the paddock) were randomly selected and a quadrat with dimensions of 0.5 × 0.5 m^2^ was placed. For TC00, using the same quadrat, the sampling points (nine in total) were placed on four endpoints located by walking serpentine fashion (zig-zag) over the entire 2 ha paddock. To evaluate the height of the grass, five points were chosen within the quadrat, which were measured from the base to the highest part of the grass, without stretching, with the help of a 100 cm graduated ruler. Subsequently, the grass was cut at ground level with a sickle, placed in identified plastic bags, and transported to the laboratory to separate the morphological components (leaf, stem, and dead matter) and then weighed individually on a Braunker model YP200 digital scale [16].

The forage samples were dried at 60°C for 72 h, and the percentage of dry matter was calculated by difference. Concomitantly, grass cover (%) in each treatment was recorded. For this procedure, a 1-m^2^ quadrat was randomly distributed in each paddock, and a visual estimate of the surface occupied by the African star grass and other grasses, and the bare soil was made [17].

### 2.6. Climate Data

The climatic data of the study site were obtained from 2 weeks before and throughout the experiment through the National Meteorological Service database, and daily measurements of ambient temperature (°C) and rainfall (millimeters) were recorded. Relative humidity (RH) data was obtained through the Weather Channel mobile application. The geographical area is classified as having a hot and humid climate. Three climatic seasons can be distinguished: rainy (June–September), winter (October–January), and dry (February–May). According to the data recorded by the CEIEGT and INEGI meteorological stations [14], the rainy season has a temperature range from 15°C to 27°C, with a precipitation of 714.7 mm, and a RH of 90% to 95%. The winter season is distinguished by temperatures from 9°C to 23°C, rainfall of 190.2 mm, and RH ranging from 30% to 90%; finally, the dry period is identified by presenting temperatures from 11°C to 29°C, rainfall of 150.1 mm, and a RH of 20%–80%. The climate formula according to García [18] based on Köeppen's classification is Af(m)w^”^e′g, or hot and humid climate with rain all year round, intrasummer drought, extreme in temperature, and Ganges-type temperature in March.

### 2.7. Statistical Analysis

The data obtained from tick counts were evaluated through descriptive analysis [19] and thereafter subjected to tests for normality and homogeneity of variances using the Anderson–Darling, Shapiro–Wilk, and Kolmogorov–Smirnov tests [20], which did not show a normal distribution, nor equal variances. For this reason, counts were transformed to log10(count +1) [21], and its variance was analyzed with mixed models that had the treatment (CT00, RT30, and RT45), breed (Brahman, and Brahman × Holstein crosses), and season (dry, rainy, and winter), and the interactions among these factors as fixed effects. The heifer within treatment and breed was the subject (random effects) [21] Previous analysis showed that coat color was not significant on counts, and also, that the interactions with the other factors of the model could not be estimated, so it was decided not including it in the model. The covariance structures: variance components (VC), compound symmetry (CS), first-order autoregressive (AR (1)), and Toeplitz (TOEP) were compared, selecting that with the lowest value of the Akaike's information criterion corrected by the number of degrees of freedom of the model (AIC_C_). The CS covariance structure was the most likely to be the correct one. The least squares means of the log transformed counts, for the main fixed effects of treatments, breeds, and the interaction treatment^∗^breed were compared with pairwise *t* tests only if the corresponding main effects and interactions in the model were significant (*p* > 0.05) or highly significant (*p* > 0.01) [22]. Transformed back counts are presented after reconversion with the formula 10^log10(count)^ − 1. The possible associations of the heifer's coat color, air temperature, RH, and rainfall with tick load was assessed with Spearman's correlation [19].

## 3. Results

Table 1 shows the results of the Type 3 test of fixed effects from the analysis of variance. The main effects of treatment, season, and the interaction treatment × season were highly significant (*p* < 0.0001, *p* < 0.0001) and significant (*p* < 0.0002) upon counts. The breed and treatment^∗^breed interaction effects were significant with *p* = 0.0119 and *p* = 0.0071, respectively. The interactions between season^∗^breed (*p* = 0.2359) and treatment^∗^season^∗^breed (*p* = 0.1923) were not significant (Table 1).

Since the treatment^∗^season and the treatment^∗^breed interactions were significant, we compared the three treatments within each season and within each breed group.  Table 2 shows the least squares mean tick load. Regardless of season, heifers in RT30 had more ticks than the other two treatments (*p* = 0.0001). The interaction occurred because the differences between the treatment counts varied in magnitude depending on the season or on the breed or cross.

### 3.1. *R. microplus* Infestations Under Grazing Systems

**Figure 1:**
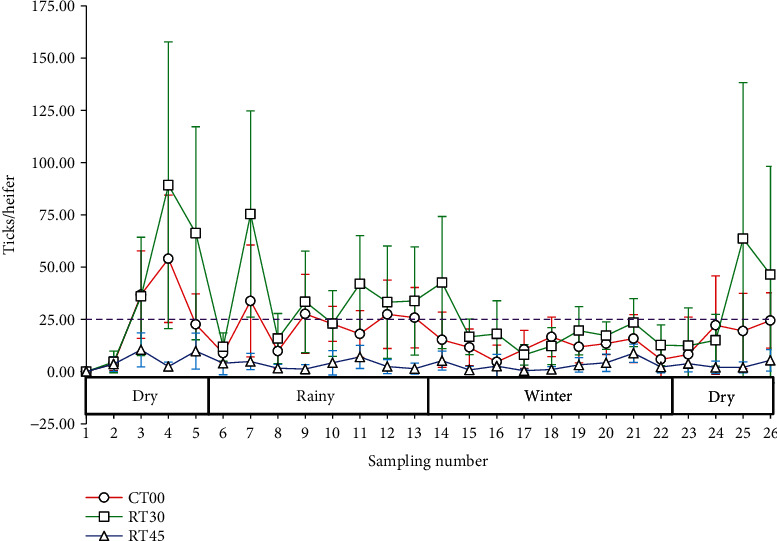
Population dynamics of *Rhipicephalus microplus* (>4.5 mm) under continuous grazing (CT00) and rotational grazing with 30 and 45 days of rest (RT30 and RT45) in Brahman calves and their crosses with Holstein, for 1 year. The dates for counting days are in Table 1. The first and last counting days dates were 15 April 2022 and 21 March 2023, respectively. The purple dotted line located at 25 ticks/heifer marks the lower limit above which the population can be considered a peak.

A total of 26 *R. microplus* tick counts were performed during the study year. In the first two counts, no difference was observed between the treatments, and the infestations in all groups were very low (Table 3). Subsequently, in 19 of 26 counts, the TR45 group presented fewer ticks than the other groups (*p* < 0.005) (Table 3; Figure 1). The animals with the highest averages of tick counts were identified in the RT30 group (*p* < 0.005), followed by animals in the CT00 group (Table 3).

The accumulated parasite load at the end of the experiment was 7720 ticks for RT30, 4690 for CT00, and 944 ticks for RT45. Three animals in the RT30 and RT45 groups harbored 50.5% (3,896 ticks) and 48.5% (458 ticks) of the infestations (cumulative counts), respectively. In CT00, three animals concentrated 45.8% (2142 ticks) of the infestations.

### 3.2. Parasitic Dynamics of *R. microplus*

Tick infestation levels varied throughout the study in all treatments (Figure 1). However, similar patterns were found between the RT30 and CT00 groups. In the RT30 group, five peaks of infestation by *R. microplus* were observed during the year: the first in the dry season, the second and third in the rainy season, the fourth during the winter season, and a fifth peak before the end of the study, in the dry season (Figure 1). No peaks of high infestations were distinguished in the RT45 group throughout the year.

During the study, temperature, RH, and rainfall per month are presented in Table 4. Regardless of treatment, the tick burden was not correlated with rainfall, RH, or temperature (*p* > 0.05). The animal's fur color and the number of ticks did not correlate either (*p* > 0.05).

### 3.3. Effects of Rotational Grazing on Pasture Characteristics

Pasture characteristics during (CT00) and before and after calves grazing (RT30 and RT45) are presented in Table 5. Throughout the year, the greatest amount of available biomass and forage height was observed in T45, followed by T30 and C00. After grazing, less grass cover was found in the RT45 treatment, whose percentage ranged from 40.5% when the calves came out, although the grass was higher (Table 5).

It is important to consider the reduction of forage quality as the rest period increased from 30 to 45 days. On the other hand, stocking density (heads/paddock/day) increased from 73.3 to 106.5 calves for 3 days in RT30 and RT45, respectively. However, the degree of use of stems + leaves were 25.38% for RT30 and 34.30% for RT45, suggests that the low utilizations lead to a high degree of selectivity that might offset possible negative effects of forage age of regrowth.

## 4. Discussion

The nonparasitic phase of *R. microplus* is one of the stages where control methods could be applied to help mitigate animal infestations, since a large part of the biological cycle of this tick takes place in pastures [10]. In the tropics and subtropics, free grazing over large areas of land is the main form of feeding for bovines, which favors the conditions for the encounter phase between tick and animal host. In this sense, various authors have mentioned that paddock rotation (with pasture rest periods) may be a feasible alternative to control tick infestations in animals [9, 13, 23, 24]. However, to have significant control of *R. microplus* in a given region during the year, it is necessary to establish adequate grazing rotation times, since some factors such as environmental and geographic conditions, cattle breeds, and type of pastures between zones can be decisive in the success of this method.

In the present investigation, a significant reduction in infestations by *R. microplus* ticks was obtained in Brahman calves and their crosses with periods of 45 days of rest from the pastures, while animals under a system with 30 days of rest show an increase in ticks infesting calves. These results indicate that a short rest period in the pastures does not reduce the parasite load in cattle, which coincides with Nicaretta et al. [12] and Cruz-González et al. [9], who reported that rotational grazing with rest periods of 20 and 30 days, respectively, are not sufficient to reduce *R. microplus* loads in cattle. It has been hypothesized that short periods of rest would cause that, when the animals enter the paddocks, the viability of the *R. microplus* larvae is found in an adequate time, promoting parasitation [9]. This could be explained considering the dynamics of the free-living phase of ticks in tropical and subtropical regions, where it has been reported that, in some pastures, *R. microplus* tick larvae can reach their highest activity at 48 days [3], considering from the detachment of the engorged tick to the hatching of larvae (42 days) and their maturation (6 days). Therefore, in the present study, if we consider that the RT30 calves rotate every 30 days and a second rotation of paddocks would add a total of 60 days and, according to [3], the pastures will still maintain 12-day-old larvae with an adequate capacity to infest animals that enter the pasture. Unlike the rest periods of 45 days, where in the first round of rotation, there would be very young larvae, and in the second rotation, the larvae would add 42 days of age beyond the optimal age and, therefore, a decreased infestation capacity due to energy expenditure and dehydration [25]. Studies under similar conditions using F1 animals (Holstein × Zebu) [9] have shown that, in the tropics, a 45-day rest on pastures significantly reduces tick loads in cattle, where the age of the larvae was considered the main factor affecting the infestations. Other factors that might influence the effect of pasture rest times against ticks are the seasonality, which influences pasture growth, the sun exposure of parasites due to variation in the vegetation cover, the precipitation, temperature, and humidity, which modify the abiotic niche of *R. microplus* larvae and eggs, affecting their development [3, 5, 26, 27].

The height of the pasture before (51.6 ± 19.4 cm) and after (31.4 ± 13.6 cm) grazing in the RT45 group was higher than that of the other treatments. Likewise, soil cover after grazing in RT45 was significantly (*p* < 0.0001) lower by 17.5% units than in RT30, which could indicate that the ticks in the pastures of this group were weakening by starvation/desiccation for being unprotected from direct sun exposure and high temperatures, affecting their survival and viability. Otherwise, the grass of the RT30 group, after grazing, maintained a greater coverage and quantity of leaves, which could be a factor that protected tick oviposition, egg incubation, hatching, and larval maturation from climatic conditions [27, 28], increasing the success of its biological cycle and its ability to parasitize cattle.

Each region has different environmental conditions and management of pastures and animals are different. There are places where periods of more than 98 days are necessary to maintain “tick-free pastures” [12, 26]. In addition, the instantaneous stocking rate on each treatment should be considered, since the higher the animal density, the lesser the movement area of the animals and, therefore, the chances of an increase in trampling of the animals during grazing may destroy the sites where the ticks oviposited, which would lead to their death or a decrease in the percentage of oviposition and/or hatching [29].

The main effect of breed (Brahman and Zebu–Holstein crosses) and its interaction with treatment were not significant (*p* = 0.1774 and *p* = 0.7103, respectively). In other words, as experimental groups, the crossed heifers were like the Brahmans in tick loads, regardless of the grazing treatment, the seasons, or their interactions. Therefore, our results suggest the necessity of including breeds resistant or resilient to ticks in integral control programs aimed at tick load reductions [30]. To reach this goal, one must identify animals with the capacity to reduce tick loads naturally. There were no effects of breed on the log 10-transformed counts. On the three treatments, the three heifers with the largest accumulated counts comprised 49%, 49%, and 45% for T00, T30, and T45, respectively. However, the accumulated loads were 2309, 3795, and 422 ticks for the same treatments. Nevertheless, using percentages could be a misleading approach to select which animals leave the herd for good. The 49% of RT30 was calculated as 3795/7720^∗^100, while the 45% of RT45 was obtained from 422/944^∗^100. The percentwise comparison between RT30 vs. RT45 leads to the idea that they are equal, but the absolute numbers implicate a different situation, as RT30 had 3795/422 = 8.99 times more ticks than RT45. The comparison is useless as tick loads are not the same and may not have the same effects on animals and, furthermore, if load thresholds have not been established. On the positive side, one can say that the removal of animals highly susceptible to infestation by *R. microplus*, and the selective treatment with chemical acaricides only at infested animals would mean a decrease in tick loads and economic cost in the medium to long term. On the other hand, the behavior of herd leaders should also be taken into consideration. Those animals, generally mature and healthy cows, are individuals that stand out for their ability to lead the movement of the group and are usually the first to enter and explore the pastures [31]. This behavior implies that tick larvae would adhere to them in greater numbers than to other individuals. It would be interesting to elucidate if the leading animals of the experimental herds were the ones with highest cattle tick loads, particularly under the rotational systems.

The three animals on each treatment that had the highest accumulated tick counts did not belong to the same breeds or crosses. On T00, there were two 3/4 Zebu–1/4 Holstein and one Brahman; on RT30, there were one 3/4 Zebu–1/4 Holstein, one 7/8 Zebu–1/8 Holstein, and one 5/8 Holstein–3/8 Zebu; and on RT45, there were one 7/8 Zebu–1/8 Holstein and two Brahman. Except for the 3/4 Holstein–1/4 Zebu, the remaining animals are resistant/resilient to ticks. In the present case, the treatment and the season (environment) were more relevant factors than the genetic one. Nonetheless, our being a small sample of animals, it is inadequate to make inferences about the real importance of the breeds/crosses in determining tick loads in cattle.

The population dynamics of *R. microplus* are influenced by multiple factors, such as the availability of hosts and their races, geographic region, climate, and management practices [4, 9, 13, 26]. In the present study, a modulation of the *R. microplus* populations was observed at different times of the year, where five population peaks were manifested in RT30, four for CT00, and three small peaks for RT45. Tick populations in bovines have shown constant growth in the last four decades, with different peaks that depend largely on their seasonality, microclimate [28], and changes in the environment [4]. Studies carried out in recent years in the tropics suggest that *R. microplus* can reach several infestation peaks (three to four) in cattle throughout the year [24, 28, 32]. In fact, the presence of five peaks has recently been reported in different neotropical regions, attributed to climate and management practices [3, 9]. In this report, no correlation was found between the loads of *R. microplus* in the animals and the climatic variables analyzed. However, a low infestation by ticks was identified during the winter, coinciding with a decrease in environmental temperature, a factor that regulates the nonparasitic phase of *R. microplus* [33], indicating a possible negative effect on tick populations.

## 5. Conclusions

Rotational grazing with 45 days of pasture rest proved to be an efficient strategy for controlling *R. microplus* in Brahman cattle and its most common crosses in the tropics of Mexico. Grazing with 30 days of pasture rest was insufficient to decrease tick loads on cattle. The characteristics of the pastures in each treatment can be an important factor in infestations. The results obtained in the present study will allow us to design and/or improve grazing management strategies for feeding cattle in tropical areas with a high incidence of *R*. *microplus* ticks.

## Figures and Tables

**Table 1:** Type 3 tests of fixed effects.

**Effect**	**Degrees of freedom**		**F** ** value**	**Pr** > **F**
	**Numerator**	**Denominator**		
Treatment	2	32.3	44.25	<0.0001
Season	2	187	52.8	<0.0001
Treatment^∗^season	4	216	5.67	0.0002
Breed	1	32.3	7.11	0.0119
Treatment^∗^breed	2	32.3	5.8	0.0071
Season^∗^breed	2	187	1.46	0.2359
Treatment^∗^season^∗^breed	4	216	1.54	0.1923

**Table 2:** Pairwise *t* test comparison between least squares means of tick counts of pasture management treatments within each season and within each breed. The comparisons were performed because the interactions between the treatment × season (*p* = 0.0002) and treatment × breed (*p* = 0.0071) were highly significant (*p* = 0.0002 and *p* = 0.0071, respectively).

**Level**	**Treatment**			**LSM ** **t** ** test treatment comparison**			**SEM**
	**CT00**	**RT30**	**RT45**	**CT00 vs. RT30**	**CT00 vs. RT45**	**RT30 vs. RT45**	
Treatment^∗^seasons (LSM of counts)							
Dry^a^	454	1193	18	0.0636	<0.0001	<0.0001	0.43
Rainy	307	722	11	0.0537	<0.0001	<0.0001	0.35
Winter	150	412	11	0.0230	<0.0001	<0.0001	0.35
Treatment^∗^breed (LSM of counts)							
Z × H^b^	545	2118	9	0.0532	<0.0001	<0.0001	0.61
Brahman	140	236	19	0.3491	0.0012	<0.0001	0.48

*Note:* LSM, least squares means transformed back from the initial log10(*x* + 1) transformation of counts; SEM, standard error.

^a^The dry, rainy, and winter seasons comprise sampling days 1–5 and 23–26; 6–13; and 14–22, respectively.

^b^The Z × H cross comprised the crosses 3/4 Brahman–1/4 Holstein, 7/8 Brahman–1/8 Holstein, and 5/8 Holstein–3/8 Brahman, respectively.

**Table 3:** Number of *R. microplus* ticks in Brahman cattle and their crosses with Holstein under three grazing modalities (CT00, RT30, and RT45) for 1 year.

**Date**	**Sampling number**	**Tick count**					
		**CT00**		**RT30**		**RT45**	
		**Means**	**SD±**	**Means**	**SD±**	**Means**	**SD±**
05/04/2022	1	0.00	0.00	0.00	0.00	0.00	0.00
19/04/2022	2	3.80	3.19	4.60	5.17	3.60	3.50
03/05/2022	3	36.80	20.94	36.00	28.32	10.40	8.10
17/05/2022	4	54.00	30.48	89.20	68.58	2.40	2.07
31/05/2022	5	22.60	14.61	66.20	50.97	9.80	8.66
14/06/2022	6	8.80	4.13	11.80	6.76	4.00	5.50
28/06/2022	7	33.80	26.77	75.40	49.40	4.80	3.91
12/07/2022	8	9.80	8.40	15.80	11.98	1.60	1.84
26/07/2022	9	27.60	18.92	33.40	24.30	1.20	2.15
09/08/2022	10	22.80	8.39	23.00	15.73	4.20	5.85
23/08/2022	11	18.00	11.20	42.00	23.09	7.00	5.52
06/09/2022	12	27.40	16.36	33.20	26.92	2.40	3.37
20/09/2022	13	25.80	14.44	33.80	25.86	1.40	2.67
04/10/2022	14	15.20	13.21	42.60	31.62	5.20	4.54
18/10/2022	15	11.60	8.78	16.60	8.49	0.80	1.69
01/11/2022	16	4.60	8.11	18.00	15.92	2.60	5.58
15/11/2022	17	10.60	9.14	8.00	4.90	0.40	0.84
29/11/2022	18	16.60	9.43	12.20	8.82	1.00	1.41
13/12/2022	19	11.80	7.63	19.60	11.54	3.20	3.43
27/12/2022	20	13.40	5.25	17.20	6.55	4.20	4.16
10/01/2023	21	15.80	11.45	23.40	11.51	8.80	4.34
24/01/2023	22	5.80	8.30	12.60	9.75	2.20	2.74
07/02/2023	23	8.20	17.80	12.40	18.13	3.80	3.94
21/02/2023	24	22.20	23.56	15.00	12.41	2.00	2.98
07/03/2023	25	19.40	18.04	63.60	74.67	2.00	2.67
21/03/2023	26	24.44	13.26	46.40	51.84	5.40	5.17

Abbreviation: SD = standard deviation.

**Table 4:** Rainfall, relative humidity, and temperature averages in the CEIEGT, from April 2022 to March 2023.

**Month**	**Rainfall (mm)**	**RH (%)**	**Temperature (°C)**
April	5.2	74	27
May	6.9	71	30
June	180.8	82	28
July	83.7	80	29
August	72.2	86	30
September	338.0	84	27
October	5.2	81	25
November	138.2	83	24
December	56.0	79	22
January	24.9	76	23
February	48.5	75	22
March	136.9	78	26

**Table 5:** Characteristics of the pastures in height, cover, and biomass (stem, leaf, and dead matter) of the grazing systems (CT00, RT30, and RT45).

**Pasture variables**	**CT00**			**RT30**						**RT45**					
	**Every 28 days**			**Before grazing**			**After grazing**			**Before grazing**			**After grazing**		
	**Mean**	**SD**	**n**	**Mean**	**SD**	**n**	**Mean**	**SD**	**n**	**Mean**	**SD**	**n**	**Mean**	**SD**	**n**
Height, cm	24.8	16.6	148	41.5	17.0	129	29.7	14.8	132	51.6	19.4	111	31.4	13.6	111
Cover, %	52.1	18.1	99	84.8	12.6	102	58.1	15.0	102	76.5	12.1	72	40.6	15.7	72
Dry matter content, %	30.7	6.3	140	35.3	8.3	121	33.2	9.3	128	29.8	6.6	108	29.5	8.1	105
Biomass, kg/ha															
Total	3299	1507	140	4889	1822	121	4011	1380	129	5160	1855	108	3971	1952	105
Stems	706	442	140	1221	829	121	891	679	129	1645	1142	108	1210	817	105
Leaves	1162	599	140	1541	737	121	1170	536	129	1655	748	108	958	734	105
Senescent matter	1431	897	140	2127	1099	121	1950	932	129	1860	1042	108	1803	1328	105

*Note:* CT00 is continuous grazing, RT30 is rotational grazing with 3-day grazing and 30-day recovery, and RT45 is rotational grazing with 3-day grazing and 45-day recovery period.

## Data Availability

The database and their statistical analyses are available upon reasonable request.
